# Bridging Food Webs, Ecosystem Metabolism, and Biogeochemistry Using Ecological Stoichiometry Theory

**DOI:** 10.3389/fmicb.2017.01298

**Published:** 2017-07-12

**Authors:** Nina Welti, Maren Striebel, Amber J. Ulseth, Wyatt F. Cross, Stephen DeVilbiss, Patricia M. Glibert, Laodong Guo, Andrew G. Hirst, Jim Hood, John S. Kominoski, Keeley L. MacNeill, Andrew S. Mehring, Jill R. Welter, Helmut Hillebrand

**Affiliations:** ^1^Department of Environmental and Biological Sciences, University of Eastern Finland Kuopio, Finland; ^2^Agriculture and Food, Commonwealth Scientific and Industrial Research Organisation, Adelaide SA, Australia; ^3^Institute for Chemistry and Biology of the Marine Environment, University of Oldenburg Oldenburg, Germany; ^4^Stream Biofilm and Ecosystem Research, Ecole Polytechnique Fédérale de Lausanne Lausanne, Switzerland; ^5^Department of Ecology, Montana State University, Bozeman MT, United States; ^6^School of Freshwater Sciences, University of Wisconsin-Milwaukee, Milwaukee WI, United States; ^7^University of Maryland Center for Environmental Science, Cambridge MD, United States; ^8^The Hirst Lab, Organismal Biology, School of Biological and Chemical Sciences, Queen Mary University of London London, United Kingdom; ^9^Centre for Ocean Life, National Institute for Aquatic Resources, Technical University of Denmark Copenhagen, Denmark; ^10^Department of Evolution, Ecology, and Organismal Biology, Aquatic Ecology Laboratory, The Ohio State University, Columbus OH, United States; ^11^The Kominoski Lab, Department of Biological Sciences, Florida International University, Miami FL, United States; ^12^Department of Ecology and Evolutionary Biology, Cornell University, Ithaca NY, United States; ^13^Scripps Institution of Oceanography, University of California, San Diego, La Jolla CA, United States; ^14^Department of Biology, St. Catherine University, Minneapolis MN, United States; ^15^Helmholtz-Institute for Functional Marine Biodiversity Oldenburg, Germany

**Keywords:** nutrient dynamics, trophic interactions, energy transfer, ecosystem function, carbon quality, element cycling, ecological stoichiometry

## Abstract

Although aquatic ecologists and biogeochemists are well aware of the crucial importance of ecosystem functions, i.e., how biota drive biogeochemical processes and vice-versa, linking these fields in conceptual models is still uncommon. Attempts to explain the variability in elemental cycling consequently miss an important biological component and thereby impede a comprehensive understanding of the underlying processes governing energy and matter flow and transformation. The fate of multiple chemical elements in ecosystems is strongly linked by biotic demand and uptake; thus, considering elemental stoichiometry is important for both biogeochemical and ecological research. Nonetheless, assessments of ecological stoichiometry (ES) often focus on the elemental content of biota rather than taking a more holistic view by examining both elemental pools and fluxes (e.g., organismal stoichiometry *and* ecosystem process rates). ES theory holds the promise to be a unifying concept to link across hierarchical scales of patterns and processes in ecology, but this has not been fully achieved. Therefore, we propose connecting the expertise of aquatic ecologists and biogeochemists with ES theory as a common currency to connect food webs, ecosystem metabolism, and biogeochemistry, as they are inherently concatenated by the transfer of carbon, nitrogen, and phosphorous through biotic and abiotic nutrient transformation and fluxes. Several new studies exist that demonstrate the connections between food web ecology, biogeochemistry, and ecosystem metabolism. In addition to a general introduction into the topic, this paper presents examples of how these fields can be combined with a focus on ES. In this review, a series of concepts have guided the discussion: (1) changing biogeochemistry affects trophic interactions and ecosystem processes by altering the elemental ratios of key species and assemblages; (2) changing trophic dynamics influences the transformation and fluxes of matter across environmental boundaries; (3) changing ecosystem metabolism will alter the chemical diversity of the non-living environment. Finally, we propose that using ES to link nutrient cycling, trophic dynamics, and ecosystem metabolism would allow for a more holistic understanding of ecosystem functions in a changing environment.

## Introduction

**FIGURE 1:**
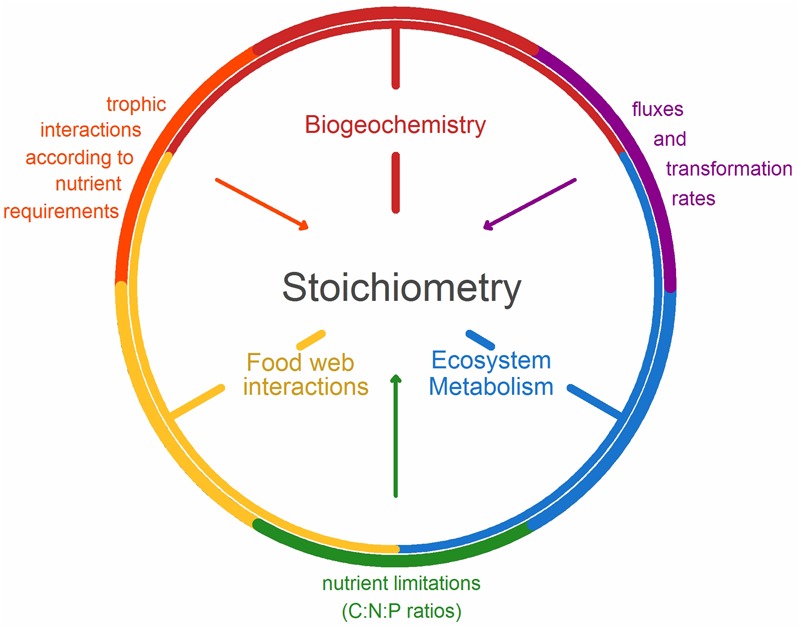
Conceptual framework demonstrating the connection between biogeochemistry, food web interactions, ecosystem metabolism, and stoichiometry. Biogeochemistry and food webs are linked through trophic interactions according to nutrient requirements between trophic levels, food webs, and ecosystem metabolism according to the nutrient limitations (C:P or C:N ratios), and ecosystem metabolism and biogeochemistry through fluxes and transformation rates.

Aquatic ecologists and biogeochemists are well aware of the importance of biologically mediated ecosystem functions in driving biogeochemical cycling and its feedback (**Figure 1**). The magnitude of ecosystem fluxes and stoichiometric constraints on biogeochemical processes are determined by turnover of elements, including the most commonly studied, carbon (C), nitrogen (N), phosphorus (P). These basal resources can be governed by ecosystem metabolism, where the balance of gross primary production (GPP) and ecosystem respiration (ER) dictate net ecosystem production (NEP). In freshwater aquatic ecosystems, when GPP exceeds ER (NEP > 0) the ecosystem is autotrophic and when ER > GPP (NEP < 0), it is heterotrophic indicating a reliance on imported C inputs, often of terrestrial origin, for respiration (Lovett et al., 2006). In other words, the biological processes of production, respiration, and excretion can drive biogeochemical cycles, therefore making it critical to understand how the elements (e.g., C, N, and P) required for these processes are coupled.

Chemical diversity in aquatic ecosystems is enormously high (Santos et al., 2008; Cai and Guo, 2009; Singer et al., 2012) and is a result of the high variety of metabolic pathways and abiotic reactions in the water column and sediment. Biological diversity can affect biogeochemical diversity, e.g., phytoplankton composition shapes the structure and functioning of the microbial loop by controlling dissolved organic matter (DOM) composition (Grossart et al., 2007; Murray et al., 2007; Passow et al., 2007; Pete et al., 2010), and thus the respective transformations and fluxes. As groups of organisms differ in terms of their elemental composition and turnover ratios, changes in the diversity of organisms are likely to affect the stoichiometry and patterns of different biogeochemical transformations and thus the flux of major elements. Scott et al. (2012) demonstrated that bacterial stoichiometry can provide a biogeochemical “set point” around which environmental variation is regulated from bottom-up controls. Furthermore, heterotrophic bacteria assemblages can have flexible and dynamic stoichiometric requirements, allowing for tight coupling and negative feedback between the bacterial requirements and the resource stoichiometry (Godwin and Cotner, 2015). Capps and Flecker (2013) showed that the growth of an introduced population of P-rich armored catfish significantly changed stream nutrient dynamics by altering nutrient storage and remineralization rates. This shows that changes in species composition can alter N and P cycling and C sequestration, producing large-scale effects on element fluxes and biogeochemical cycles.

Autotrophs and heterotrophs drive C and nutrient cycling in aquatic ecosystems. Therefore, the balance of GPP and ER controls the source and quality of C, thereby creating the basis for food webs (Marcarelli et al., 2011). Autochthonous material is usually higher in C quality than allochthonous material (Findlay et al., 1986) although terrestrial allochthonous material can have higher C:N and N:P ratios (Lennon and Pfaff, 2005). In terms of ecosystem metabolism, when NEP > 0 (i.e., autotrophic), the bulk C source is likely of autochthonous origin, and hence of high quality. When an ecosystem is heterotrophic (i.e., NEP < 0), allochthonous material subsidizes ER, indicating the potential for a lower quality C source (Findlay et al., 1986; Zhou et al., 2016). Most aquatic ecosystems are heterotrophic throughout the year (Vannote et al., 1980; Battin et al., 2008; Hoellein et al., 2013), resulting in high-flux, low-quality subsidies driving freshwater ecosystem dynamics (Marcarelli et al., 2011). However, the production of autochthonous material, including any window of autotrophy, is a key flux. The autochthonous fluxes are often low in quantity, but of high-quality, which support food webs and affect ecosystem processes (Marcarelli et al., 2011). The extent to which allochthonous material incorporated into food webs is less understood for many stream ecosystems (Marcarelli et al., 2011; Bartels et al., 2012; Collins et al., 2015; but see Wallace et al., 1999 for forest streams). Additionally, ecosystem metabolism is inherently linked to nutrient (N and/or P), and C-cycling; yet, given this fact, there are few studies which have coupled ecosystem metabolism to nutrient cycling (Hall and Tank, 2003; Webster et al., 2003; Hall et al., 2013; Hoellein et al., 2013), C-spiraling (Hall et al., 2016), or both nutrient and organic C egestion and assimilation (Hall et al., 2003).

Changes in environmental drivers, such as temperature or nutrient availability, can alter biodiversity and influence the transformation and fluxes of organic matter and nutrients in these ecosystems. Temperature has strong effects on growth rates and the physiology of phytoplankton (Eppley, 1972; Karentz and Smayda, 1984; Butterwick et al., 2005) and can also influence protist mean cell size (Atkinson et al., 2003; Forster et al., 2013), nutrient uptake rates (Senft et al., 2008), N metabolism and cell stoichiometry (Lomas and Glibert, 1999; Montagnes and Franklin, 2001; Litchman et al., 2010), and ER (Yvon-Durocher et al., 2012). Such effects on autotrophic and heterotrophic producers likely affect consumers directly. Thus, trophic interactions, food web structure and mutualistic networks can result in cascading effects on ecosystem metabolism or vice versa. Many studies take a biogeochemical approach (mainly in streams) focused on individual elements (e.g., Meyer and Likens, 1979; Triska et al., 1984; Mulholland et al., 2000) or on the effect of ratios on the flux of single elements (Dodds et al., 2004; Schade et al., 2011). Martiny et al. (2013) showed that strong latitudinal patterns exist in the elemental ratios of marine plankton and organic matter and others have examined the relationship between phytoplankton diversity and particulate ratios across biogeochemical gradients (Salter et al., 2014; Rembauville et al., 2015). In general, most studies from aquatic ecosystems focus on the cycling of N or P as these are the nutrients most likely to limit primary production. However, Elser et al. (2007) and Harpole et al. (2011) pointed towards the prevalence of multiple nutrient limitation to primary production in most aquatic and terrestrial habitats. Further, Boersma and Elser (2006) and Glibert et al. (2013) underscored the importance of nutrients not just at the limiting end of the availability spectrum, but across the continuum from limitation to excess. Combining biogeochemical models with ecological stoichiometry (ES), and thus using traceable mass balance relationships, can be a way to describe and understand the complex interactions and feedbacks more completely (Franklin et al., 2011).

Here, we discuss the many ways in which ES links food webs, ecosystem metabolism and biogeochemistry, thus influencing stocks and fluxes of key elements (cf. Glibert et al., 2011). The fate of multiple elements in ecosystems requires consideration of elemental stoichiometry for both biogeochemical and ecological research. Based on a literature search (**Table 1**), a large number of studies included any of the three terms—food webs, ecosystem metabolism, and biogeochemistry—together with ES, but only eight studies used ES in connection to all three terms. ES has the potential to be a concept unifying flux-oriented biogeochemistry, ecosystem metabolism, and population-oriented ecology, but so far only a few studies have achieved this (Reiners, 1986). For example, Hall et al. (2003) linked N production and demand, ecosystem metabolism, and snail production using ES. By assuming that net primary production was 50% of GPP, and based on the expected C:N ratio of 14:1 of C to N fixation, the authors estimated that these snails ingested 75% of daily GPP and that excretion of snails was estimated 65% of total NH_4_ demand. The authors concluded that this invasive snail dominated C and N fluxes, despite very high GPP and N demand. In this case, ES provided a quantitative framework for linking inorganic nutrients, stream metabolism, and secondary production.

**Table 1:** Numbers of publications (Web of Science searching all databases, accessed March 2017) including key words for one of the research fields (metabolism, stoichiometry, food web, or biogeochemistry) and combinations of these key words.

Keyword	Number of publications
Metabolism	7,480,534
Stoichiometry	98,372
Food web	34,496
Biogeochemistry	11,441
Metabolism + stoichiometry	15,166
Metabolism + food web	4,950
Metabolism + biogeochemistry	1,620
Food web + stoichiometry	521
Food web + biogeochemistry	454
Biogeochemistry + stoichiometry	278
Metabolism + food web + biogeochemistry	79
Metabolism + food web + stoichiometry	111
Metabolism + biogeochemistry + stoichiometry	66
Food web + biogeochemistry + stoichiometry	39
Metabolism + food web + biogeochemistry + stoichiometry	8

Studies of ES have often focused on the elemental content of specific types of organisms rather than combining biological with physical and chemical drivers of element fluxes, including ecosystem metabolism. Changes in the diversity of key taxa can have major impacts on a range of biogeochemical transformations and overall fluxes. For example, both increased light and the introduction of the guppy (*Poecilia reticulata*) increased N fluxes to some invertebrate functional feeding groups (Collins et al., 2016). The advantage of combining these fields of expertise is that effects of multiple changes of more than one parameter can be investigated. For example, when considering multiple nutrient limitations, the flux of more than one element should be considered—a task that can be achieved by combining biogeochemical approaches using ES. Investigating the interactions of temperature and nutrients by combining ES (Sterner and Elser, 2002) and metabolic theory of ecology (Brown et al., 2004) will improve the understating of microbial and ecosystem ecology (Hall et al., 2010) on different levels of organization (individuals, populations, communities, food webs, ecosystem; see reviews by Cross et al., 2015; Vanni and McIntyre, 2016). Diet-induced metabolic plasticity contributes to variation in metabolic allometry, at least at small scales of body size due to the greater respiratory response of smaller species to altered diets (Jeyasingh, 2007). Moorthi et al. (2016) showed that unifying ES and metabolic theory allows us to predict production and trophic transfer in a marine planktonic food web. Changes in nutrient loading have become a major concern among all scales of organization and can have strong impacts on biogeochemical cycles (Falkowski et al., 2000). Results from Manning et al. (2016) indicate that changes in basal resource stoichiometry can occur due to effects on either autotrophic (e.g., biofilm) or heterotrophic microbial communities, resulting in diminished stream consumer biodiversity related to either heterotrophic or autotrophic food web pathways. Many environmental changes, such as climate warming, eutrophication, acidification, and CO_2_ alter absolute nutrient supply and likely nutrient ratios (e.g., Boyd and Hutchins, 2012; Glibert et al., 2014). Therefore, a combined approach including metabolic theory and ES is valuable for assessing the possible effects of environmental changes (Hessen et al., 2013).

## Empirical Assessments

In the following section, we exemplify how food web interactions, ecosystem metabolism, and biogeochemistry can use ES theory to integrate from microbial to ecosystem-scale processes through a series of case studies. The examples are derived from a special session at the 2016 Association for the Sciences of Limnology and Oceanography (ASLO) meeting in Santa Fe, NM, United States, with the aim to merge the fields of biogeochemistry, food webs and ecosystem metabolism by using ES as a common theoretical framework. Using the following research highlights, we convey the depth and range of approaches which have been applied, that merge these disciplines, which are conceptualized in our model (**Figure 1**). In our first case study, ES links a general trait of metabolism (body mass dependence) to trophic interactions and biogeochemistry by demonstrating changes in resource transport and N:O ratios. Secondly, ES demonstrates the interactions between trophic dynamics of benthic aquatic invertebrates and two large-scale biogeochemical fluxes. Thirdly, the addition of trace elements to the traditional C:N:P ratios improves the understanding of altered trophic interactions and nutrient fluxes. And then in the subsequent two examples, the N:P loads shift over time, allowing for the proliferation of invasive species which further impact that quality of carbon and N:P availability. Furthermore, the sixth case study uses ES to demonstrate how changes to N:P alters ecosystem metabolism through enhanced microbial respiration rates and food web interactions. Finally, the interaction between biogeochemistry with regard to changing temperature is quantified using ES and the impact on ecosystem metabolism. The diversity of our examples illustrates the potential strength of this approach for understanding relationships among and across trophic levels, including biogeochemical interactions as well as direct and indirect effects.

### A New Model to Explain the Body Mass Scaling of Diverse Biological Rates in Aquatic Invertebrates

Body size is a “master trait” that affects all vital rates, including feeding, reproduction, excretion and metabolism (Kleiber, 1932, 1961; Schmidt-Nielsen, 1984; Hirst et al., 2014). Understanding what drives the body mass dependence of such a wide diversity of rates is of fundamental biological importance, indeed, this has been a much-debated topic over the last century. Recent work has explored body mass scaling exponents of metabolic rates within planktonic species (Hirst et al., 2014; Glazier et al., 2015) in order to better appreciate what controls these terms, and ultimately to better predict these rates for species and communities. These authors tested two groups of theories that predict the body-mass dependence of metabolism, those built upon internal transport networks (including the Metabolic Theory of Ecology; West et al., 1999; Savage et al., 2008; Banavar et al., 2010), and those based on a Surface Area model [a reapplication of Rubner’s surface dependent model of heat exchange in endotherms (Rubner, 1883), but more broadly applied to the influx and efflux of materials and energy]. Importantly, many zooplankton change body shape as they grow, while also using significant proportions of their body surface for the exchange of materials. While the major geometric scaling theories produce rather similar predictions when shape does not change over ontogeny (i.e., they are isomorphic), the predictions from these two groups of theory diverge starkly when organisms increasingly flatten or elongate in shape over ontogeny. These shape changes result in a reduction in the predicted scaling exponents of many resource transport model, but increase the predicted scaling exponent for the Surface Area dependent model. While the mass-scaling of respiration has been shown to correlate with body surface enlargement in many pelagic invertebrates (Hirst et al., 2014; Glazier et al., 2015), Hirst et al. (2016) predicted that body-mass scaling exponents for rates of soluble N excretion (b_N_) should also then relate to the degree of body-shape change during growth. They tested this hypothesis using literature data on b_N_ for pelagic invertebrates across five different phyla, and found that b_N_ is significantly positively correlated with predicted surface area enlargement, whilst also co-varying with the mass-scaling of respiration rate (b_R_). Indeed, intraspecific differences between b_N_ and b_R_ values have revealed there are shifts in the ratio of O_2_-consumed to N-excreted over ontogeny. This suggests that changes in the relative anabolism and catabolism of proteins and lipids over development, may cause these consumption-excretion ratios to change too. In conclusion, diverse pelagic invertebrates, that dominate vast open water ecosystems, therefore appear to falsify the predictions of general metabolic scaling theories built upon resource-transport networks, while supporting predictions of surface-area dependent theory. Furthermore, ontogenetic variation in ratios of O_2_ consumed to N excreted of these species, may not only provide insight into the developmental metabolism, but also the stoichiometry of ecological systems, including, for example, seasonal changes in N-budgets that are linked to pelagic animal life cycles.

### Enhancement of Carbon Dioxide, Methane, and Nitrous Oxide Flux by Invertebrates

Aquatic ecosystems can be sources of greenhouse gases (GHG), a process that is strongly controlled by the availability of C, N, and P, which can stimulate emission of nitrous oxide (N_2_O), methane (CH_4_), and carbon dioxide (CO_2_) (Cao et al., 1996; Burgin et al., 2013; Nisbet et al., 2014; Deemer et al., 2016). However, mounting evidence suggests that benthic aquatic invertebrates such as midge larvae (Diptera: Chironomidae), snails (Gastropoda), and aquatic worms (Oligochaeta and Polychaeta) can enhance the emissions of GHG through high N excretion rates, by creating anoxic microenvironments within their guts, and through bioturbation and bioirrigation of surrounding sediments (Kristensen et al., 1991; Nielsen et al., 2004; Figueiredo-Barros et al., 2009; Stief et al., 2009; Heisterkamp et al., 2010; Nogaro and Burgin, 2014; Poulsen et al., 2014; Hölker et al., 2015; Mehring et al., 2017).

A large portion of the CH_4_ produced in freshwater and marine sediments that is not released by ebullition is oxidized to CO_2_ or assimilated by methanotrophic bacteria (Bastviken et al., 2008). Some species of midge larvae and zooplankton have been shown to assimilate methane-derived C through consumption of methanotrophic bacteria (Deines et al., 2007), as evidenced by exceptionally low stable isotopic ratios (δ^13^C as low -64‰ for midge larvae; Jones et al., 2008). It is still unclear if differences in faunal isotopic ratios among aquatic ecosystems can be consistently linked to differences in ecosystem function, or if the effects of methanotroph consumption by invertebrates are substantial enough to influence emissions across the air–water interface of lakes and wetlands. For example, Kajan and Frenzel (1999) observed that both production and oxidation of CH_4_ were enhanced in chironomid burrows in rice paddies, but there was no net effect on benthic CH_4_ flux. The feeding activity of bacterivorous zooplankton such as Cladocera has been shown to suppress methanotrophic activity in laboratory mesocosms (Kankaala et al., 2007), but this has not yet been demonstrated to affect CH_4_ fluxes at large scales. Conversely, bioturbation is a non-consumptive mechanism by which benthic fauna may influence CH_4_ flux, which has been demonstrated in manipulative laboratory studies (Figueiredo-Barros et al., 2009) but has yet to be linked to differences in faunal stoichiometry.

While much work is needed to further elucidate the enhancement of microbial metabolic pathways and GHG flux by aquatic invertebrates, previous studies have demonstrated enhancement of GHG flux by invertebrates under highly controlled conditions in laboratories. An assessment of the effects of mixed assemblages (and likely resulting in a wide range of nutrient stoichiometry) under variable conditions is important to our understanding of faunal influence on GHG fluxes in aquatic ecosystems. Since taxa such as Tubificinae have been shown to enhance GHG flux (Nogaro and Burgin, 2014; Mehring et al., 2017) and also to reach high densities in eutrophic aquatic environments (Devine and Vanni, 2002), invertebrate enhancement of GHG emissions from aquatic ecosystems may be linked both to anthropogenically induced nutrient loading and resulting shifts in aquatic community structures. Given the variable environmental conditions in mixed biotic assemblages outside of controlled laboratory conditions, the degree to which the effects of invertebrates and their corresponding C:N:P can be detected relative to other drivers of GHG flux in field settings requires further investigation.

### Including Trace Elements for a Holistic Stoichiometric Approach in Food Webs

ES is an important framework for examining paired biogeochemical processes; however, ES studies in both terrestrial and aquatic systems are biased toward C, N, and P while trace elements are often neglected (Sterner and Elser, 2002). Recently, Kaspari and Powers (2016) argued the importance of expanding traditional models of co-limitation to include all 25 of life’s building elements. Including non-essential trace elements is also crucial to a holistic stoichiometric approach (MacNeill et al., 2016). Arsenic (As), mercury (Hg), selenium (Se) and other non-essential trace elements have been well studied individually (Boening, 2000; Farag et al., 2003; Schaller et al., 2010; Walters et al., 2015), but their pairings with other, more common elements have less frequently been evaluated (but see Wang et al., 2013). Integrating trace elements, their interactions with each other and their interactions with C, N, and P into studies of ES will provide a more complete picture of elemental cycling in ecosystems (Wang et al., 2013). The toxic trace element As can alter both ecosystem structure and function: In terms of ecosystem structure, As contamination decreases stream invertebrate abundance and diversity (Chaffin et al., 2005). Functionally, As affects cycling of common (N and P) stream nutrients (Lottig et al., 2007; Rodriguez Castro et al., 2015; MacNeill et al., 2016). In freshwaters, P is usually in the form of phosphate (PO_4_^3-^), which shares the same chemical structure as arsenate (AsO_4_^3-^), the most common form of As in oxygenated freshwaters (Button et al., 1973; Schaller et al., 2010). Consequently, As can be taken into bacterial, algal, and animal cells in place of P and decouple oxidative- and photo-phosphorylation, hindering energy production (Finnegan and Chen, 2012). Cells are less able to distinguish between As and P when P is low relative to As (Rodriguez Castro et al., 2015) and in particular when total P is less than ∼50 μg/L, as is the case in a majority of freshwaters (Villanueva et al., 2000; Binkley et al., 2004; Hall et al., 2013). Recently published research shows that As metabolism by the algae *Chlorella vulgaris* depends on the relative amount of P, which determines both uptake of P and the dominant metabolite excreted by cells (Baker and Wallschläger, 2016).

In addition to the interchangeability of As and P, the cycles of N and P are intimately linked (Cross et al., 2005; Schade et al., 2011). Because the cycles of N and P are so intertwined, it is likely that the As cycle is linked to the N cycle through P. Toxic effects of As tend to be greater in P limited environments (Rodriguez Castro et al., 2015) and P limitation depends on relative N availability (Tessier and Raynal, 2003; Schade et al., 2011; Rodriguez Castro et al., 2015). Therefore, linkages with N may explain why previous studies have not satisfactorily resolved how As affects P uptake (Pringle, 1991; Lottig et al., 2007; Hoellein et al., 2012). MacNeill et al. (2016) found evidence that ambient dissolved N:P, rather than P concentration alone or relative As:P, influences the amount of As removed from the water column by biofilm (assemblages of bacteria, algae, and fungi growing on rocks) uptake. The relative N:P dissolved in water as a driver of As uptake by biofilms has implications for the amount of As, metabolized by, retained in, and transferred through food webs. Therefore, expanding the framework of ES to include trace elements is important to understand their relationships with common elements and their effects on ecosystem functioning.

### Applying Ecological Stoichiometry and Biogeochemistry Together to Understand Changes in Aquatic Food Webs and Invasive Species

**FIGURE 2:**
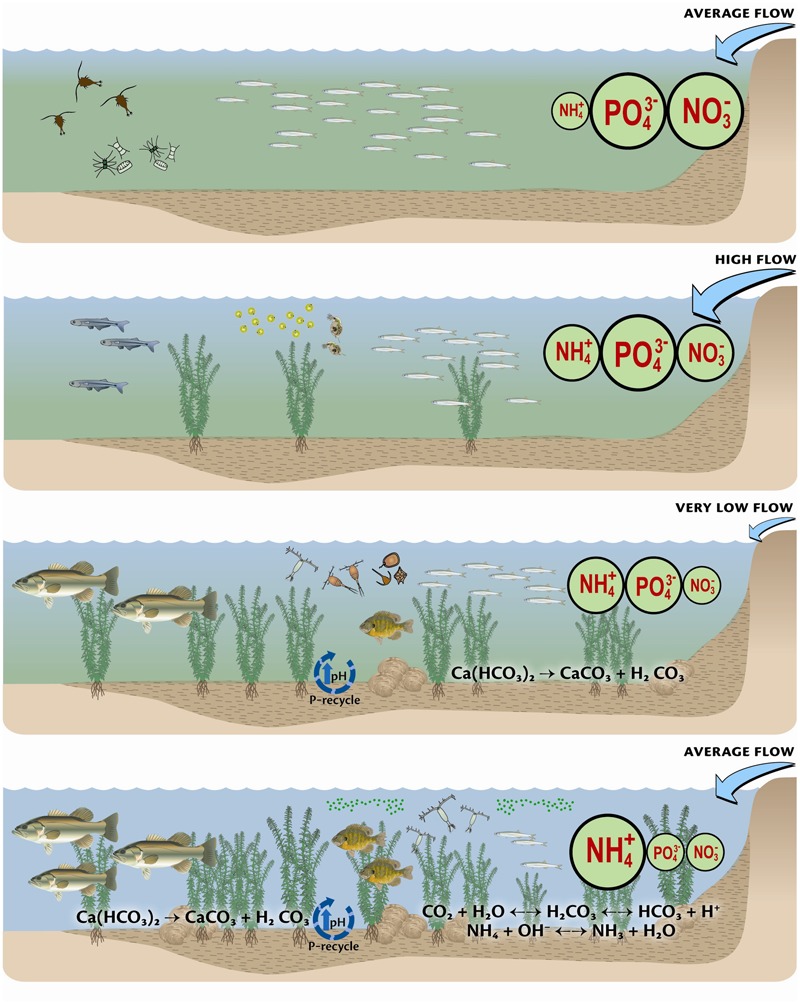
Conceptual depiction of the change over time in major nutrients, flow, dominant biogeochemical processes, and the food web of the Bay Delta. The first panel represents the period from 1975 to ∼1982, when flow was low, and diatoms and *Eurytemora* were the dominant phytoplankton and zooplankton, respectively, and smelt were common. The second panel represents the period from ∼1982 to 1986 when flow was high, and NH_4_^+^ was increasing. During this period the food web began to change. Under very low flow conditions, depicted by the third panel, and representing ∼1987 to 1995, the NH_4_^+^ load was high but PO_4_^3-^ began to decrease. The food web also began to change significantly, with changes in the dominant phytoplankton and zooplankton, increasing abundance of macrophytes, increased importance of sediment nutrient processes, and increase in piscivores. Finally, post 1995, NH_4_^+^ loads remain high, while PO_4_^3-^ loads are proportionately low. Sediment biogeochemical processes are of increasing importance in nutrient processing, macrophyte production is important and omnivorous fish have increased. At the microbial level, *Microcystis* is more common and the zooplankton is dominated by cyclopoids, e.g., *Limnoithona*. Reproduced from Glibert (2012) with permission of the publisher.

ES, together with biogeochemistry has been applied to understanding invasive species and changes to aquatic food webs in the San Francisco Bay Delta (Glibert et al., 2011; Glibert, 2012). In this ecosystem, the food web has changed significantly over the past decades, from phytoplankton to fish. Using 30 years of records of nutrient loads and concentrations and abundances of phytoplankton, zooplankton, macroinvertebrates, and fish it was shown that changes in ratios of N and P, together with changes in N form, have been significant drivers of changes in the food web (**Figure 2**). Members of different trophic levels were found to have different correlations with N and P, as did taxa within trophic levels. These patterns were consistent with the premise that the fish community shifted to species that were proportionately more P-rich over time as N and P ratios increased due to substantial increases in N loading and reductions in P. The patterns were also consistent with increased importance of a benthic food web following reductions in P loading. Changes in external nutrient loads also drove changes in biogeochemical fluxes at the sediment water interface, leading to increasing abundance of macrophytes, clams, and of the toxic algae *Microcystis*, along with more omnivorous fish fueled by a benthic food web. The picture that has emerged of this ecosystem is one where changes in the food web are now understood to follow the conceptual model of stoichiometry, and not purely stochastic events. Previously considered one of the most heavily invaded estuaries in the world, it is now clear that environmental changes, including nutrient ratios and concentrations, interact with vectors of invasion to enhance their success.

### The Role of Invasive Quagga Mussels in Affecting Dissolved Organic Matter in Lake Michigan

Invasive quagga mussels (*Dreissena rostriformis bugensis*) have caused unprecedented ecological and environmental changes in Lake Michigan. Declines in primary production, fish biomass, and turbidity as well as significant changes to food web structure, phytoplankton composition, and nutrient cycling pathways have all occurred as a result of the introduction of quagga mussels (Bunnell et al., 2006; Cuhel and Aguilar, 2013; Lin and Guo, 2016). As efficient ecosystem engineers, quagga mussels voraciously filter pelagic particulate matter and excrete/egest nutrients in the benthos resulting in significant alterations to water column and benthic chemistry (Schindler and Scheuerell, 2002; Madenjian et al., 2015). Specifically, nutrients and organic matter that served as an energy source for forage fish have been intercepted by quagga mussels and sequestered in the benthos. Therefore, quantifying the specific mechanisms and pathways by which invasive quagga mussels have altered organic C and nutrient cycling are needed to understand the response of the Lake Michigan ecosystem to these non-indigenous bivalves. In the absence of particulate organic matter, which has become scarce in the water column of Lake Michigan, quagga mussels have been shown to efficiently remove materials in the dissolved and colloidal phase (DeVilbiss and Guo, 2017). For example, laboratory incubations have demonstrated the ability of quagga mussels to efficiently remove material as small as 0.5 μm, indicating their potential to directly uptake DOM in the water column. Quagga mussels also directly excrete DOM, with smaller mussels excreting at a significantly higher rate than larger mussels. Excreted DOM had unique chromophoric and fluorescent properties characteristic of protein-like materials, a colloidal size spectrum centered at 1–5 kDa, low TOC/TDN ratios (1.1 ± 0.1) but higher TDN/TDP ratios (33 ± 4) and was predominately composed of structural (refractory) polysaccharides. These results indicated that excreted DOM was chemically altered not only in regards to C molecules, but in N:P ratios as well. Based on initial estimations, only around 11% of consumed organic C was excreted/egested by quagga mussels, indicating that quagga mussels may be a potential sink for organic matter as well as a CO_2_ source via metabolism.

### Applying ES to Understand Effects of Added Nutrients on Microbial to Ecosystem-Scale Carbon Loss

Understanding effects of nutrient addition on microbial to ecosystem-scale metabolic processes is essential to expanding theoretical predictions of elemental limitation among ecosystems (Elser et al., 2007). Ecosystems that are autotrophic are generally co-limited by N and P (Elser et al., 2007), whereas donor-controlled, detritus-based ecosystems are dominated by heterotrophic consumers whose responses to added nutrients depend on the stoichiometry of detrital resources (Manning et al., 2015). Added N and P both accelerate C loss in detritus-based streams through enhanced organic matter breakdown and export (Benstead et al., 2009; Rosemond et al., 2015; Manning et al., 2016), as well as through substrate-specific and whole-stream ER (Suberkropp et al., 2010; Kominoski et al., 2017). Litter breakdown rates are constrained by microbial nutrient limitation (both N and P) at low-to-moderate concentrations through changes in litter C:N and C:P stoichiometry (Kominoski et al., 2015; Manning et al., 2015). These collective findings emphasize the importance of microbial processes on ecosystem C loss and the potential for long-term vulnerability to sustained C losses with sustained or increased N and P availability (Alexander and Smith, 2006), which ultimately can be linked to nutrient stoichiometry.

Long-term studies of nutrient enrichment in forest streams show declines in ecosystem-scale C. Studies of added N and P in streams of the Coweeta Long Term Ecological Research Program in the southern Appalachians, United States, illustrate that nutrients increase C loss through enhanced microbial respiration rates and invertebrate feeding activities (Benstead et al., 2009; Suberkropp et al., 2010). Increasing N and P concentrations while maintaining N:P ratios can accelerate in-stream biological process that result in up to a 50% reduction in residence time of terrestrial C (Rosemond et al., 2015). Declines in organic matter standing stocks and increases in associated respiration rates with nutrient enrichment, appear to be driven more by N than P. Nutrient enrichment can alter the relationships between N and P supply ratio and ecosystem-level processes. For example, prior to nutrient enrichment whole-stream ER in Coweeta streams was higher at lower N:P, but during enrichment ER increased with increasing N:P (Kominoski et al., 2017). Increased heterotrophy from microbial to ecosystem-scales can occur at concentrations of N and P that are now common among pristine and human-impacted ecosystems (Alexander and Smith, 2006).

### Combining Metabolic Ecology and Ecological Stoichiometry to Develop a Mechanistic Understanding of How Temperature Influences Freshwater Metabolism

A central challenge for ecologists is to understand how climate warming will influence GPP and ER, due to the central role these processes play in structuring food web production and C and nutrient cycles (Peterson et al., 2001; Raymond et al., 2013; Hotchkiss et al., 2015). The combined frameworks of metabolic ecology and ES offer promise for developing a mechanistic understanding of how temperature influences freshwater metabolism (Sterner and Elser, 2002; Sibly et al., 2012). Yet, more explicit consideration of the coupling between metabolic theory and ES is required (Sterner, 2004; Cross et al., 2015). A growing literature suggests that temperature dependences of ecosystem processes may diverge strongly from predictions, particularly when temperature influences—or is associated with—changes in resource supply (Anderson-Teixeira et al., 2008; Valett et al., 2008; Yvon-Durocher et al., 2012; Huryn et al., 2014; Welter et al., 2015). A better mechanistic understanding of how temperature and nutrients interact to influence metabolism will likely improve these predictive models.

Model ecosystems, that are natural, can provide a powerful tool for quantifying these mechanisms at the ecosystem level. The Hengill geothermal area in Iceland represents one such natural laboratory for examining how temperature influences the structure and function of stream ecosystems (O’Gorman et al., 2012, 2014) by allowing a combination of field surveys, stream-side channel experiments, and whole-stream temperature manipulations. Recent experiments have discovered that temperature dependences (measured as apparent “activation energies”; Brown et al., 2004) for GPP and ER were 6.5- and 2.7-fold higher, respectively, than predicted by Metabolic Theory; interestingly, these relationships were similar to the temperature dependency of N_2_-fixation (Welter et al., 2015), suggesting a strong interaction between temperature and nutrient supply. The stronger than expected temperature dependencies for GPP and ER likely resulted from N-limitation of production at low temperatures and release from N-limitation at warm temperatures by N_2_-fixation and the addition of “new” N. In addition, these studies showed that N limitation was further alleviated by a temperature-induced increase in N use efficiency (Williamson et al., 2016). A similar increase in flux-based N use efficiency was found in a survey of natural geothermal streams, as well as a whole-stream warming experiment in this Icelandic catchment (Hood et al., unpublished data). Taken together, these results promise that a better understanding of the interactive effects of temperature and nutrients on organisms and elemental fluxes can be used to develop a strong mechanistic understanding of how climate warming will influence river metabolism.

## Summary and Outlook

**FIGURE 3:**
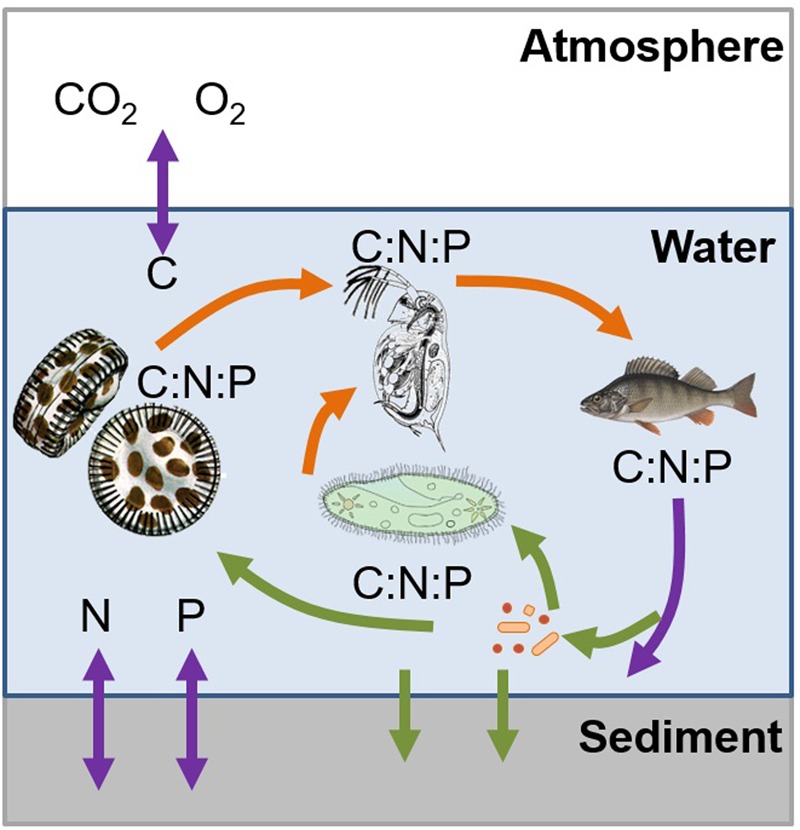
Example demonstrating how ecological stoichiometry can be used to link food web interactions, ecosystem metabolism, and biogeochemistry in a system, as they are inherently linked by the transfer of carbon, nitrogen, and phosphorous through biotic and abiotic nutrient transformation and fluxes. The trophic interactions (orange arrows) are occurring based on the nutrient requirements which are limited by the available nutrients (green arrows) as they are transferred and transformed (purple arrows) between the atmosphere, water column, and sediment. The colors of arrows indicate the processes described in **Figure 1**.

The examples described above demonstrate that ES can be a useful tool for linking food web interactions, ecosystem metabolism, and biogeochemistry (**Figure 1**). As demonstrated in the previous examples, altered nutrient concentrations, ratios or fluxes, either through anthropogenic or system-induced pathways, results in changes in ecosystem functioning (**Figure 3**). By increasing nutrient concentrations, organic matter decomposition increases and results in overall C loss in aquatic ecosystems. Furthermore, these increased nutrient concentrations may induce a shift toward favorable conditions for invasive species to persist (Glibert, 2015), or shifts toward community structures that enhance microbial metabolism and GHG emissions. Our examples show that it is not only the absolute nutrient concentrations that create these conditions; rather it is both, the concentrations and the ratio of the nutrients that can alter or drive one process over the other. Furthermore, organisms can alter the composition of chemical compounds (as illustrated by the quagga mussel example altering the DOM diversity in a lake), resulting in an overall change to the ecosystem. While we have begun to explore the role of macronutrients, the relative contribution of micronutrients, especially how they interact with other nutrients (as in the case of As and P), is less understood. Such interactions between macro- and micronutrients can potentially alter the stoichiometric balance and thus should be included in future studies. Temperature and nutrient turnover are inherently linked and the examples presented here point to the links between temperature and nutrient cycling and thus the effect of temperature on nutrient ratios.

Along with the above examples, we have demonstrated the current state-of-the-art approaches, which link food web interactions, ecosystem metabolism, and biogeochemistry along the following concepts and processes (**Figure 1**):

1. Changing biogeochemistry affects trophic interactions and ecosystem processes by altering the elemental ratios of key species and assemblages.
  ◦ The stoichiometry of biogeochemical processes links the biological turnover rates of major elements, such that changes in biodiversity result in changes in mineral nutrient ratios in biogeochemical pools and fluxes.
2. Changing trophic dynamics influences the transformation and fluxes of matter across environmental boundaries.
  ◦ Through biogeochemical pathways, change in a focal group of organisms has propagating consequences on the functioning of other compartments and on the metabolism of aquatic ecosystems.
  ◦ Trophic interactions, food web structure, and mutualistic networks will result in cascading effects on ecosystem metabolism or vice versa.
3. Changing ecosystem metabolism will alter the chemical diversity of the non-living environment.
  ◦ The alteration of metabolic processes in aquatic ecosystems affects the transformation and fluxes of inorganic and organic matter.
  ◦ The molecular diversity of non-living organic matter is functionally linked to the diversity of organisms. Chemical diversity influences and is influenced by shifts in biodiversity.

The future goal is to use the theory of ES as a common currency to connect food web interactions, ecosystem metabolism, and biogeochemistry as they are inherently linked by the transfer of C, N, and P through biotic and abiotic nutrient transformations and fluxes in order to improve our understanding of aquatic ecosystem functioning. Given the future projections of climate change for increasing temperature and anthropogenic nutrient loading, ES can be essential to understand and predict the links between food web interactions, biogeochemistry, and ecosystem metabolism and elucidate the controls which underpin the processes that ultimately drives nutrient and energy fluxes in aquatic ecosystems.

## Author Contributions

NW and MS contributed equally to this manuscript. NW, MS, and AU conceived the manuscript. All authors contributed substantially to the manuscript, revised it for important intellectual content, approved the final version, and agreed to be accountable for all aspects of the work.

## Conflict of Interest Statement

The authors declare that the research was conducted in the absence of any commercial or financial relationships that could be construed as a potential conflict of interest.

## References

[B1] Alexander R. B. Smith R. A. (2006). Trends in the nutrient enrichment of US rivers during the late 20th century and their relation to changes in probable stream trophic conditions. *Limnol. Oceanogr.* 51 639–654. 10.4319/lo.2006.51.1_part_2.0639

[B2] Anderson-Teixeira K. J. Vitousek P. M. Brown J. H. (2008). Amplified temperature dependence in ecosystems developing on the lava flows of Mauna Loa. Hawai’i. *Proc. Natl. Acad. Sci. U.S.A.* 105 228–233. 10.1073/pnas.0710214104 18156366 PMC2224191

[B3] Atkinson D. Ciotti B. J. Montagnes D. J. (2003). Protists decrease in size linearly with temperature: ca. 2.5% C- 1. *Proc. R. Soc. Lond. B Biol. Sci.* 270 2605–2611. 10.1098/rspb.2003.2538 PMC1691543 14728784

[B4] Baker J. Wallschläger D. (2016). The role of phosphorus in the metabolism of arsenate by a freshwater green alga, *Chlorella vulgaris*. *J. Environ. Sci.* 49 169–178. 10.1016/j.jes.2016.10.002 28007172

[B5] Banavar J. R. Moses M. E. Brown J. H. Damuth J. Rinaldo A. Sibly R. M. (2010). A general basis for quarter-power scaling in animals. *Proc. Natl. Acad. Sci. U.S.A.* 107 15816–15820. 10.1073/pnas.1009974107 20724663 PMC2936637

[B6] Bartels P. Cucherousset J. Steger K. Eklöv P. Tranvik L. J. Hillebrand H. (2012). Reciprocal subsidies between freshwater and terrestrial ecosystems structure consumer resource dynamics. *Ecology* 93 1173–1182. 10.1890/11-1210.1 22764503

[B7] Bastviken D. Cole J. J. Pace M. L. Van de Bogert M. C. (2008). Fates of methane from different lake habitats: connecting whole-lake budgets and CH4 emissions. *J. Geophys. Res. Biogeosci.* 113 G02024 10.1029/2007JG000608

[B8] Battin T. J. Kaplan L. A. Findlay S. Hopkinson C. S. Marti E. Packman A. I. (2008). Biophysical controls on organic carbon fluxes in fluvial networks. *Nat. Geosci.* 1 95–100. 10.1038/ngeo101

[B9] Benstead J. P. Rosemond A. D. Cross W. F. Wallace J. B. Eggert S. L. Suberkropp K. (2009). Nutrient enrichment alters storage and fluxes of detritus in a headwater stream ecosystem. *Ecology* 90 2556–2566. 10.1890/08-0862.1 19769133

[B10] Binkley D. Ice G. G. Kaye J. Williams C. A. (2004). Nitrogen and phosphorus concentrations in forest streams of the United States. *J. Am. Water Resour. Assoc.* 40 1277–1291. 10.1111/j.1752-1688.2004.tb01586.x

[B11] Boening D. W. (2000). Ecological effects, transport, and fate of mercury: a general review. *Chemosphere* 40 1335–1351. 10.1016/S0045-6535(99)00283-0 10789973

[B12] Boersma M. Elser J. J. (2006). Too much of a good thing: on stoichiometrically balanced diets and maximal growth. *Ecology* 87 1325–1330. 10.1890/0012-9658(2006)87[1325:TMOAGT]2.0.CO;2 16761610

[B13] Boyd P. W. Hutchins D. A. (2012). Understanding the responses of ocean biota to a complex matrix of cumulative anthropogenic change. *Mar. Ecol. Prog. Ser.* 470 125–135. 10.3354/meps10121

[B14] Brown J. H. Gillooly J. F. Allen A. P. Savage V. M. West G. B. (2004). Toward a metabolic theory of ecology. *Ecology* 85 1771–1789. 10.1890/03-9000

[B15] Bunnell D. B. Madenjian C. P. Claramunt R. M. (2006). Long-term changes of the Lake Michigan fish community following the reduction of exotic alewife (*Alosa pseudoharengus*). *Can. J. Fish. Aquat. Sci.* 63 2434–2446. 10.1139/f06-132

[B16] Burgin A. J. Lazar J. G. Groffman P. M. Goldb A. J. Kellogg D. Q. (2013). Balancing nitrogen retention ecosystem services and greenhouse gas disservices at the landscape scale. *Ecol. Eng.* 56 26–35. 10.1016/j.ecoleng.2012.05.003

[B17] Butterwick C. Heaney S. I. Talling J. F. (2005). Diversity in the influence of temperature on the growth rates of freshwater algae, and its ecological relevance. *Freshw. Biol.* 50 291–300. 10.1111/j.1365-2427.2004.01317.x

[B18] Button D. Dunker S. S. Morse M. (1973). Continuous culture of *Rhodotorula rubra*: kinetics of phosphate-arsenate uptake, inhibition, and phosphate-limited growth. *J. Bacteriol.* 113 599–611. 4690960 10.1128/jb.113.2.599-611.1973 PMC285271

[B19] Cai Y. Guo L. (2009). Abundance and variation of colloidal organic phosphorus in riverine, estuarine, and coastal waters in the northern Gulf of Mexico. *Limnol. Oceanogr.* 54 1393–1402. 10.4319/lo.2009.54.4.1393

[B20] Cao M. Marshall S. Gregson K. (1996). Global carbon exchange and methane emissions from natural wetlands: application of a process-based model. *J. Geophys. Res. Atmos.* 101 14399–14414. 10.1029/96JD00219

[B21] Capps K. A. Flecker A. S. (2013). Invasive fishes generate biogeochemical hotspots in a nutrient-limited system. *PLoS ONE* 8:e54093 10.1371/journal.pone.0054093 PMC3546933 23342083

[B22] Chaffin J. L. Valett H. M. Webster J. R. Schreiber M. E. (2005). Influence of elevated as on leaf breakdown in an Appalachian headwater stream. *J. North Am. Benthol. Soc.* 24 553–568. 10.1899/04-058.1

[B23] Collins S. M. Kohler T. J. Thomas S. A. Fetzer W. W. Flecker A. S. (2015). The importance of terrestrial subsidies in stream food webs varies along a stream size gradient. *Oikos* 125 674–685. 10.1111/oik.02713

[B24] Collins S. M. Thomas S. A. Heatherly T. II MacNeill K. L. Leduc A. O. López-Sepulcre A. (2016). Fish introductions and light modulate food web fluxes in tropical streams: a whole-ecosystem experimental approach. *Ecology* 97 3154–3166. 10.1002/ecy.1530 27870030

[B25] Cross W. F. Benstead J. P. Frost P. C. Thomas S. A. (2005). Ecological stoichiometry in freshwater benthic systems: recent progress and perspectives. *Freshw. Biol.* 50 1895–1912. 10.1111/j.1365-2427.2005.01458.x

[B26] Cross W. F. Hood J. M. Benstead J. P. Huryn A. D. Nelson D. (2015). Interactions between temperature and nutrients across levels of ecological organization. *Glob. Change Biol.* 21 1025–1040. 10.1111/gcb.12809 25400273

[B27] Cuhel R. L. Aguilar C. (2013). Ecosystem transformations of the Laurentian Great Lake Michigan by nonindigenous biological invaders. *Mar. Sci.* 5 289–320. 10.1146/annurev-marine-120710-100952 22809179

[B28] Deemer B. R. Harrison J. A. Li S. Beaulieu J. J. DelSontro T. Barros N. (2016). Greenhouse gas emissions from reservoir water surfaces: a new global synthesis. *Bioscience* 66 949–964. 10.1093/biosci/biw117 PMC7425809 32801383

[B29] Deines P. Bodelier P. L. Eller G. (2007). Methane-derived carbon flows through methane-oxidizing bacteria to higher trophic levels in aquatic systems. *Environ. Microbiol.* 9 1126–1134. 10.1111/j.1462-2920.2006.01235.x 17472629

[B30] DeVilbiss S. E. Guo L. (2017). Excretion of organic matter and nutrients from invasive quagga mussels and potential impact on carbon dynamics in Lake Michigan. *J. Great Lakes Res.* 43 79–89. 10.1016/j.jglr.2017.03.002

[B31] Devine J. A. Vanni M. J. (2002). Spatial and seasonal variation in nutrient excretion by benthic invertebrates in a eutrophic reservoir. *Freshw. Biol.* 47 1107–1121. 10.1046/j.1365-2427.2002.00843.x

[B32] Dodds W. K. Gido K. Whiles M. R. Fritz K. M. Matthews W. J. (2004). Life on the edge: the ecology of Great Plains prairie streams. *BioScience* 54 205–216. 10.1641/0006-3568(2004)054[0205:LOTETE]2.0.CO;2

[B33] Elser J. J. Bracken M. E. Cleland E. E. Gruner D. S. Harpole W. S. Hillebrand H. (2007). Global analysis of nitrogen and phosphorus limitation of primary producers in freshwater, marine and terrestrial ecosystems. *Ecol. Lett.* 10 1135–1142. 10.1111/j.1461-0248.2007.01113.x 17922835

[B34] Eppley R. W. (1972). Temperature and phytoplankton growth in the sea. *Fish. Bull.* 70 1063–1085.

[B35] Falkowski P. Scholes R. J. Boyle E. Canadell J. Canfield D. Elser J. (2000). The global carbon cycle: a test of our knowledge of earth as a system. *Science* 290 291–296. 10.1126/science.290.5490.291 11030643

[B36] Farag A. M. Skaar D. Nimick D. A. MacConnell E. Hogstrand C. (2003). Characterizing aquatic health using salmonid mortality, physiology, and biomass estimates in streams with elevated concentrations of arsenic, cadmium, copper, lead, and zinc in the Boulder River watershed, Montana. *Trans. Am. Fish. Soc.* 132 450–467. 10.1577/1548-8659(2003)132<0450:CAHUSM>2.0.CO;2

[B37] Figueiredo-Barros M. P. Caliman A. Leal J. J. Bozelli R. L. Farjalla V. F. Esteves F. A. (2009). Benthic bioturbator enhances CH4 fluxes among aquatic compartments and atmosphere in experimental microcosms. *Can. J. Fish. Aquat. Sci.* 66 1649–1657. 10.1139/F09-111

[B38] Findlay S. Carlough L. Crocker M. T. Kay Gill H. Meyer J. L. Smith P. J. (1986). Bacterial growth on macrophyte leachate and fate of bacterial production. *Limnol. Oceanogr.* 31 1335–1341. 10.4319/lo.1986.31.6.1335

[B39] Finnegan P. M. Chen W. (2012). Arsenic toxicity: the effects on plant metabolism. *Front. Physiol.* 3:182 10.3389/fphys.2012.00182 PMC3368394 22685440

[B40] Forster J. Hirst A. G. Esteban G. F. (2013). Achieving temperature-size changes in a unicellular organism. *ISME J.* 7 28–36. 10.1038/ismej.2012.76 22832346 PMC3526166

[B41] Franklin O. Hall E. K. Kaiser C. Battin T. J. Richter A. (2011). Optimization of biomass composition explains microbial growth-stoichiometry relationships. *Am. Nat.* 177 E29–E42. 10.1086/657684 21460549

[B42] Glazier D. S. Hirst A. G. Atkinson D. (2015). Shape shifting predicts ontogenetic changes in metabolic scaling in diverse aquatic invertebrates. *Proc. R. Soc. Lond. B Biol. Sci.* 282:20142302 10.1098/rspb.2014.2302 PMC4344145 25652833

[B43] Glibert P. M. (2012). Ecological stoichiometry and its implications for aquatic ecosystem sustainability. *Curr. Opin. Environ. Sustain.* 4 272–277. 10.1016/j.cosust.2012.05.009

[B44] Glibert P. M. (2015). More than propagule pressure: successful invading algae have physiological adaptations suitable to anthropogenically changing nutrient environments. *Aquat. Ecosyst. Health Manag.* 18 334–341.

[B45] Glibert P. M. Fullerton D. Burkholder J. M. Cornwell J. C. Kana T. M. (2011). Ecological stoichiometry, biogeochemical cycling, invasive species, and aquatic food webs: San Francisco Estuary and comparative systems. *Rev. Fish. Sci.* 19 358–417. 10.1080/10641262.2011.611916

[B46] Glibert P. M. Kana T. M. Brown K. (2013). From limitation to excess: the consequences of substrate excess and stoichiometry for phytoplankton physiology, trophodynamics and biogeochemistry, and the implications for modeling. *J. Mar. Syst.* 125 14–28. 10.1016/j.jmarsys.2012.10.004

[B47] Glibert P. M. Maranger R. Sobota D. J. Bouwman L. (2014). The Haber Bosch–harmful algal bloom (HB–HAB) link. *Environ. Res. Lett.* 9 105001 10.1088/1748-9326/9/10/105001

[B48] Godwin C. M. Cotner J. B. (2015). Aquatic heterotrophic bacteria have highly flexible phosphorus content and biomass stoichiometry. *ISME J.* 9 2324–2327. 10.1038/ismej.2015.34 25798755 PMC4579474

[B49] Grossart H. Engel A. Arnosti C. De La Rocha C. L. Murray A. E. Passow U. (2007). Microbial dynamics in autotrophic and heterotrophic seawater mesocosms. III. Organic matter fluxes. *Aquat. Microb. Ecol.* 49 143–156. 10.3354/ame01140

[B50] Hall E. K. Singer G. A. Kainz M. J. Lennon J. T. (2010). Evidence for a temperature acclimation mechanism in bacteria: an empirical test of a membrane-mediated trade-off. *Funct. Ecol.* 24 898–908. 10.1111/j.1365-2435.2010.01707.x

[B51] Hall R. O. Baker M. A. Rosi-Marshall E. J. Tank J. L. Newbold J. D. (2013). Solute-specific scaling of inorganic nitrogen and phosphorus uptake in streams. *Biogeosciences* 10 7323–7331. 10.5194/bg-10-7323-2013

[B52] Hall R. O. Tank J. L. (2003). Ecosystem metabolism controls nitrogen uptake in streams in Grand Teton National Park, Wyoming. *Limnol. Oceanogr.* 48 1120–1128. 10.4319/lo.2003.48.3.1120

[B53] Hall R. O. Tank J. L. Baker M. A. Rosi-Marshall E. J. Hotchkiss E. R. (2016). Metabolism, gas exchange, and carbon spiraling in rivers. *Ecosystems* 19 73–86. 10.1007/s10021-015-9918-1

[B54] Hall R. O. Tank J. L. Dybdahl M. F. (2003). Exotic snails dominate nitrogen and carbon cycling in a highly productive stream. *Front. Ecol. Environ.* 1:407–411. 10.1890/1540-9295(2003)001[0407:esdnac]2.0.co;2

[B55] Harpole W. S. Ngai J. T. Cleland E. E. Seabloom E. W. Borer E. T. Bracken M. E. (2011). Nutrient co-limitation of primary producer communities: community co-limitation. *Ecol. Lett.* 14 852–862. 10.1111/j.1461-0248.2011.01651.x 21749598

[B56] Heisterkamp I. Schramm A. de Beer D. Stief P. (2010). Nitrous oxide production associated with coastal marine invertebrates. *Mar. Ecol. Prog. Ser.* 415 1–9. 10.3354/meps08727

[B57] Hessen D. O. Elser J. J. Sterner R. W. Urabe J. (2013). Ecological stoichiometry: an elementary approach using basic principles. *Limnol. Oceanogr.* 58 2219–2236. 10.4319/lo.2013.58.6.2219

[B58] Hirst A. G. Glazier D. S. Atkinson D. (2014). Body shape shifting during growth permits tests that distinguish between competing geometric theories of metabolic scaling. *Ecol. Lett.* 17 1274–1281. 10.1111/ele.12334 25060740

[B59] Hirst A. G. Lilley M. K. S. Glazier D. S. Atkinson D. (2016). Ontogenetic body-mass scaling of nitrogen excretion relates to body surface area in diverse pelagic invertebrates: N-excretion in pelagic invertebrates. *Limnol. Oceanogr.* 62 311–319. 10.1002/lno.10396

[B60] Hoellein T. J. Bruesewitz D. A. Hamilton D. P. (2012). Are geothermal streams important sites of nutrient uptake in an agricultural and urbanising landscape (Rotorua, New Zealand)?: nutrient uptake and metabolism in geothermal streams. *Freshw. Biol.* 57 116–128. 10.1111/j.1365-2427.2011.02702.x

[B61] Hoellein T. J. Bruesewitz D. A. Richardson D. C. (2013). Revisiting Odum (1956): a synthesis of aquatic ecosystem metabolism. *Limnol. Oceanogr.* 58 2089–2100. 10.4319/lo.2013.58.6.2089

[B62] Hölker F. Vanni M. J. Kuiper J. J. Meile C. Grossart H. P. Stief P. (2015). Tube-dwelling invertebrates: tiny ecosystem engineers have large effects in lake ecosystems. *Ecol. Monogr.* 85 333–351. 10.1890/14-1160.1

[B63] Hotchkiss E. Hall R. Jr. Sponseller R. Butman D. Klaminder J. Laudon H. (2015). Sources of and processes controlling CO_2_ emissions change with the size of streams and rivers. *Nat. Geosci.* 8 696–699. 10.1038/ngeo2507

[B64] Huryn A. D. Benstead J. P. Parker S. M. (2014). Seasonal changes in light availability modify the temperature dependence of ecosystem metabolism in an arctic stream. *Ecology* 95 2826–2839. 10.1890/13-1963.1 30854634

[B65] Jeyasingh P. D. (2007). Plasticity in metabolic allometry: the role of dietary stoichiometry. *Ecol. Lett.* 10 282–289. 10.1111/j.1461-0248.2007.01023.x 17355567

[B66] Jones R. I. Carter C. E. Kelly A. Ward S. Kelly D. J. Grey J. (2008). Widespread contribution of methane-cycle bacteria to the diets of lake profundal chironomid larvae. *Ecology* 89 857–864. 10.1890/06-2010.1 18459348

[B67] Kajan R. Frenzel P. (1999). The effect of chironomid larvae on production, oxidation and fluxes of methane in a flooded rice soil. *FEMS Microbiol. Ecol.* 28 121–129. 10.1111/j.1574-6941.1999.tb00567.x

[B68] Kankaala P. Eller G. Jones R. I. (2007). Could bacterivorous zooplankton affect lake pelagic methanotrophic activity? *Fundam. Appl. Limnol.* 169 203–209. 10.1127/1863-9135/2007/0169-0203

[B69] Karentz D. Smayda T. J. (1984). Temperature and seasonal occurrence patterns of 30 dominant phytoplankton species in Narragansett Bay over a 22-year period (1959–1980). *Mar. Ecol. Prog. Ser.* 18 277–293. 10.3354/meps018277

[B70] Kaspari M. Powers J. S. (2016). Biogeochemistry and geographical ecology: embracing all twenty-five elements required to build organisms. *Am. Nat.* 188 S62–S73. 10.1086/687576 27513911

[B71] Kleiber M. (1932). Body size and metabolism. *Hilgardia* 6 315–353. 10.3733/hilg.v06n11p315

[B72] Kleiber M. (1961). *The Fire of Life: An Introduction to Animal Energetics*. New York, NY: Wiley.

[B73] Kominoski J. S. Rosemond A. D. Benstead J. P. Gulis V. Maerz J. C. Manning D. W. (2015). Low-to-moderate nitrogen and phosphorus concentrations accelerate microbially driven litter breakdown rates. *Ecol. Appl.* 25 856–865. 10.1890/14-1113.1 26214929

[B74] Kominoski J. S. Rosemond A. D. Benstead J. P. Gulis V. Manning D. W. P. (2017). Experimental nitrogen and phosphorus additions increase rates of stream ecosystem respiration and carbon loss. *Limnol. Oceanogr.* 10.1002/lno.10610 [Epub ahead of print].

[B75] Kristensen E. Hjorth Jensen M. Aller R. C. (1991). Direct measurement of dissolved inorganic nitrogen exchange and denitrification in individual polychaete (*Nereis virens*) burrows. *J. Mar. Res.* 49 355–377. 10.1357/002224091784995855

[B76] Lennon J. Pfaff L. (2005). Source and supply of terrestrial organic matter affects aquatic microbial metabolism. *Aquat. Microb. Ecol.* 39 107–119. 10.3354/ame039107

[B77] Lin P. Guo L. (2016). Dynamic changes in the abundance and chemical speciation of dissolved and particulate phosphorus across the river-lake interface in southwest Lake Michigan. *Limnol. Oceanogr.* 61 771–789. 10.1002/lno.10254

[B78] Litchman E. de Tezanos Pinto P. Klausmeier C. A. (2010). Linking traits to species diversity and community structure in phytoplankton. *Hydrobiologia* 653 15–28. 10.1007/s10750-010-0341-5

[B79] Lomas M. W. Glibert P. M. (1999). Temperature regulation of nitrate uptake: a novel hypothesis about nitrate uptake and reduction in cool-water diatoms. *Limnol. Oceanogr.* 44 556–572. 10.4319/lo.1999.44.3.0556

[B80] Lottig N. R. Maurice Valett H. Schreiber M. E. Webster J. R. (2007). Flooding and arsenic contamination: influences on ecosystem structure and function in an Appalachian headwater stream. *Limnol. Oceanogr.* 52 1991–2001. 10.4319/lo.2007.52.5.1991

[B81] Lovett G. M. Cole J. J. Pace M. L. (2006). Is net ecosystem production equal to ecosystem carbon accumulation? *Ecosystems* 9 152–155. 10.1007/s10021-005-0036-3

[B82] MacNeill K. L. Collins S. M. Encalada A. C. Kohler B. S. Thomas S. A. Rosi-Marshall E. (2016). “Arsenic controls on stoichiometry and nutrient cycling in tropical streams,” in *Proceedings of the ASLO Conference 2016* [Abstract ID:28047] Santa Fe, NM.

[B83] Madenjian C. P. Bunnell D. B. Warner D. M. Pothoven S. A. Fahnenstiel G. L. Nalepa T. H. (2015). Changes in the Lake Michigan food web following dreissenid mussel invasions: a synthesis. *J. Great Lakes Res.* 41 217–231. 10.1016/j.jglr.2015.08.009

[B84] Manning D. W. Rosemond A. D. Gulis V. Benstead J. P. Kominoski J. S. Maerz J. C. (2016). Convergence of detrital stoichiometry predicts thresholds of nutrient-stimulated breakdown in streams. *Ecol. Appl.* 26 1745–1757. 10.1890/15-1217.1 27755690

[B85] Manning D. W. Rosemond A. D. Kominoski J. S. Gulis V. Benstead J. P. Maerz J. C. (2015). Detrital stoichiometry as a critical nexus for the effects of streamwater nutrients on leaf litter breakdown rates. *Ecology* 96 2214–2224. 10.1890/14-1582.1 26405746

[B86] Marcarelli A. M. Baxter C. V. Mineau M. M. Hall R. O. (2011). Quantity and quality: unifying food web and ecosystem perspectives on the role of resource subsidies in freshwaters. *Ecology* 92 1215–1225. 10.1890/10-2240.1 21797150

[B87] Martiny A. C. Pham C. T. A. Primeau F. W. Vrugt I. A. Moore J. K. Levin S. A. (2013). Strong latitudinal patterns in the elemental ratios of marine plankton and organic matter. *Nat. Geosci.* 6 279–283. 10.1038/ngeo1757

[B88] Mehring A. S. Cook P. L. M. Evrard V. Grant S. B. Levin L. A. (2017). Pollution-tolerant invertebrates enhance greenhouse gas flux in urban wetlands. *Ecol. Appl.* 10.1002/eap.1572 [Epub ahead of print]. 28482116

[B89] Meyer J. L. Likens G. E. (1979). Transport and transformation of phosphorus in a forest stream ecosystem. *Ecology* 60 1255–1269. 10.2307/1936971

[B90] Montagnes D. J. Franklin M. (2001). Effect of temperature on diatom volume, growth rate, and carbon and nitrogen content: reconsidering some paradigms. *Limnol. Oceanogr.* 46 2008–2018. 10.4319/lo.2001.46.8.2008

[B91] Moorthi S. D. Schmitt J. A. Ryabov A. Tsakalakis I. Blasius B. Prelle L. (2016). Unifying ecological stoichiometry and metabolic theory to predict production and trophic transfer in a marine planktonic food web. *Philos. Trans. R. Soc. B Biol. Sci.* 371:20150270 10.1098/rstb.2015.0270 PMC4843692 27114573

[B92] Mulholland P. J. Tank J. L. Sanzone D. M. Wollheim W. M. Peterson B. J. Webster J. R. (2000). Nitrogen cycling in a forest stream determined by a 15N tracer addition. *Ecol. Monogr.* 70 471–493.

[B93] Murray A. E. Arnosti C. De La Rocha C. Grossart H. P. Passow U. (2007). Microbial dynamics in autotrophic and heterotrophic seawater mesocosms. II. Bacterioplankton community structure and hydrolytic enzyme activities. *Aquat. Microb. Ecol.* 49 123–141. 10.3354/ame01139

[B94] Nielsen O. I. Gribsholt B. Kristensen E. Revsbech N. P. (2004). Microscale distribution of oxygen and nitrate in sediment inhabited by *Nereis diversicolor*: spatial patterns and estimated reaction rates. *Aquat. Microb. Ecol.* 34 23–32. 10.3354/ame034023

[B95] Nisbet E. G. Dlugokencky E. J. Bousquet P. (2014). Methane on the rise—again. *Science* 343 493–495. 10.1126/science.1247828 24482471

[B96] Nogaro G. Burgin A. J. (2014). Influence of bioturbation on denitrification and dissimilatory nitrate reduction to ammonium (DNRA) in freshwater sediments. *Biogeochemistry* 120 279–294. 10.1007/s10533-014-9995-9

[B97] O’Gorman E. J. Benstead J. P. Cross W. F. Friberg N. Hood J. M. Johnson P. W. (2014). Climate change and geothermal ecosystems: natural laboratories, sentinel systems, and future refugia. *Glob. Change Biol.* 20 3291–3299. 10.1111/gcb.12602 24729541

[B98] O’Gorman E. J. Pichler D. E. Adams G. (2012). Impacts of warming on the structure and functioning of aquatic communities: individual-to ecosystem-level responses. *Adv. Ecol. Res.* 47 81–176. 10.1016/B978-0-12-398315-2.00002-8

[B99] Passow U. De La Rocha C. L. Arnosti C. Grossart H. P. Murray A. Engel A. (2007). Microbial dynamics in autotrophic and heterotrophic seawater mesocosms. I. Effect of phytoplankton on the microbial loop. *Aquat. Microb. Ecol.* 49 109–121. 10.3354/ame01138

[B100] Pete R. Davidson K. Hart M. C. Gutierreza T. Miller A. E. J. (2010). Diatom derived dissolved organic matter as a driver of bacterial productivity: the role of nutrient limitation. *J. Exp. Mar. Biol. Ecol.* 391 20–26. 10.1016/j.jembe.2010.06.002

[B101] Peterson B. J. Wollheim W. M. Mulholland P. J. Webster J. R. Meyer J. L. Tank J. L. (2001). Control of nitrogen export from watersheds by headwater streams. *Science* 292 86–90. 10.1126/science.1056874 11292868

[B102] Poulsen M. Kofoed M. V. Larsen L. H. Schramm A. Stief P. (2014). *Chironomus plumosus* larvae increase fluxes of denitrification products and diversity of nitrate-reducing bacteria in freshwater sediment. *Syst. Appl. Microbiol.* 37 51–59. 10.1016/j.syapm.2013.07.006 24054696

[B103] Pringle C. M. (1991). Geothermally modified waters surface at La Selva Biological Station, Costa Rica: volcanic processes introduce chemical discontinuities into lowland tropical streams. *Biotropica* 23 523–529. 10.2307/2388390

[B104] Raymond P. A. Hartmann J. Lauerwald R. Sobek S. McDonald C. Hoover M. (2013). Global carbon dioxide emissions from inland waters. *Nature* 503 355–359. 10.1038/nature12760 24256802

[B105] Reiners W. A. (1986). Complementary models for ecosystems. *Am. Nat.* 127 59–73. 10.1086/284467

[B106] Rembauville M. Blain S. Armand L. Quéguiner B. Salter I. (2015). Export fluxes in a naturally iron-fertilized area of the Southern Ocean – Part 2: importance of diatom resting spores and faecal pellets for export. *Biogeosciences* 12 3171–3195. 10.5194/bg-12-3171-2015

[B107] Rodriguez Castro M. C. Urrea G. Guasch H. (2015). Influence of the interaction between phosphate and arsenate on periphyton’s growth and its nutrient uptake capacity. *Sci. Total Environ.* 503–504 122–132. 10.1016/j.scitotenv.2014.06.094 25005240

[B108] Rosemond A. D. Benstead J. P. Bumpers P. M. Gulis V. Kominoski J. S. Manning D. W. (2015). Experimental nutrient additions accelerate terrestrial carbon loss from stream ecosystems. *Science* 347 1142–1145. 10.1126/science.aaa1958 25745171

[B109] Rubner M. (1883). Ueber den einfluss der korpergrosse auf stoffund kaftwechsel. *Z. Biol.* 19 535–562.

[B110] Salter I. Schiebel R. Ziveri P. Movellan A. Lampitt R. Wolff G. A. (2014). Carbonate counter pump stimulated by natural iron fertilization in the Polar Frontal Zone. *Nat. Geosci.* 7 885–889. 10.1038/ngeo2285

[B111] Santos I. R. Burnett W. C. Chanton J. Mwashote B. Suryaputra I. G. N. A. Dittmar T. (2008). Nutrient biogeochemistry in a Gulf of Mexico subterranean estuary and groundwater-derived fluxes to the coastal ocean. *Limnol. Oceanogr.* 53 705–718. 10.4319/lo.2008.53.2.0705

[B112] Savage V. M. Deeds E. J. Fontana W. (2008). Sizing up allometric scaling theory. *PLoS Comput. Biol.* 4:e1000171 10.1371/journal.pcbi.1000171 PMC2518954 18787686

[B113] Schade J. D. MacNEILL K. Thomas S. A. Camille McNeely F. Welter J. R. Hood J. (2011). The stoichiometry of nitrogen and phosphorus spiralling in heterotrophic and autotrophic streams: stoichiometry of N and P spiralling in streams. *Freshw. Biol.* 56 424–436. 10.1111/j.1365-2427.2010.02509.x

[B114] Schaller J. Weiske A. Mkandawire M. Dudel E. G. (2010). Invertebrates control metals and arsenic sequestration as ecosystem engineers. *Chemosphere* 79 169–173. 10.1016/j.chemosphere.2010.01.015 20132960

[B115] Schindler D. E. Scheuerell M. D. (2002). Habitat coupling in lake ecosystems. *Oikos* 98 177–189. 10.1034/j.1600-0706.2002.980201.x

[B116] Schmidt-Nielsen K. (1984). *Scaling: Why is Animal Size so Important?* Cambridge: Cambridge University Press 10.1017/CBO9781139167826

[B117] Scott J. T. Cotner J. B. LaPara T. M. (2012). Variable stoichiometry and homeostatic regulation of bacterial biomass elemental composition. *Front. Microbiol.* 3:42 10.3389/fmicb.2012.00042 PMC3283892 22371708

[B118] Senft W. H. Hunchberger R. A. Roberts K. E. (2008). Temperature dependence of growth and phosphorus uptake in two species of *Volvox* (Volvovales. Chlorophyta). *J. Phycol.* 17 323–329. 10.1111/j.1529-8817.1981.tb00858.x

[B119] Sibly R. M. Brown J. H. Kodric-Brown A. (2012). *Metabolic Ecology: A Scaling Approach.* Hoboken, NJ: John Wiley & Sons 10.1002/9781119968535

[B120] Singer G. A. Fasching C. Wilhelm L. Niggemann J. Steier P. Dittmar T. (2012). Biogeochemically diverse organic matter in Alpine glaciers and its downstream fate. *Nat. Geosci.* 5 710–714. 10.1038/ngeo1581

[B121] Sterner R. W. (2004). A one-resource“stoichiometry”? *Ecology* 85 1813–1816. 10.1890/03-0724

[B122] Sterner R. W. Elser J. J. (2002). *Ecological Stoichiometry: The Biology of Elements from Molecules to the Biosphere.* Princeton, NJ: Princeton University Press.

[B123] Stief P. Poulsen M. Nielsen L. P. Brix H. Schramm A. (2009). Nitrous oxide emission by aquatic macrofauna. *Proc. Natl. Acad. Sci. U.S.A.* 106 4296–4300. 10.1073/pnas.0808228106 19255427 PMC2651200

[B124] Suberkropp K. Gulis V. Rosemond A. D. Benstead J. (2010). Ecosystem and physiological scales of microbial responses to nutrients in a detritus-based stream: results of a 5-year continuous enrichment. *Limnol. Oceanogr.* 55 149–160. 10.4319/lo.2010.55.1.0149

[B125] Tessier J. T. Raynal D. J. (2003). Use of nitrogen to phosphorus ratios in plant tissue as an indicator of nutrient limitation and nitrogen saturation. *J. Appl. Ecol.* 40 523–534. 10.1046/j.1365-2664.2003.00820.x

[B126] Triska F. J. Sedell J. R. Cromack K. Gregory S. V. Michael McCorison F. (1984). Nitrogen budget for a small coniferous forest stream. *Ecol. Monogr.* 54 119–140. 10.2307/1942458

[B127] Valett H. M. Thomas S. A. Mulholland P. J. Webster J. R. Dahm C. N. Fellows C. S. (2008). Endogenous and exogenous control of ecosystem function: N cycling in headwater streams. *Ecology* 89 3515–3527. 10.1890/07-1003.1 19137956

[B128] Vanni M. J. McIntyre P. B. (2016). Predicting nutrient excretion of aquatic animals with metabolic ecology and ecological stoichiometry: a global synthesis. *Ecology* 97 3460–3471. 10.1002/ecy.1582 27912023

[B129] Vannote R. L. Minshall G. W. Cummins K. W. Sedell J. R. Cushing C. E. (1980). The river continuum concept. *Can. J. Fish. Aquat. Sci.* 37 130–137. 10.1139/f80-017

[B130] Villanueva D. Queimaliños C. Modenutti B. Ayala J. (2000). Effects of fish farm effluents on the periphyton of an Andean stream. *Arch. Fish. Mar. Res.* 48 283–294.

[B131] Wallace J. B. Eggert S. Meyer J. L. Webster J. (1999). Effects of resource limitation on a detrital-based ecosystem. *Ecol. Monogr.* 69 409–442. 10.1890/0012-9615(1999)069[0409:EORLOA]2.0.CO;2

[B132] Walters D. M. Rosi-Marshall E. Kennedy T. A. Cross W. F. Baxter C. V. (2015). Mercury and selenium accumulation in the Colorado River food web, Grand Canyon, USA: Hg and Se in the Colorado River Food Web, Grand Canyon. *Environ. Toxicol. Chem.* 34 2385–2394. 10.1002/etc.3077 26287953

[B133] Wang N. X. Li Y. Deng X. H. Miao A. J. Ji R. Yang L. Y. (2013). Toxicity and bioaccumulation kinetics of arsenate in two freshwater green algae under different phosphate regimes. *Water Res.* 47 2497–2506. 10.1016/j.watres.2013.02.034 23497978

[B134] Webster J. R. Mulholland P. J. Tank J. L. Valett H. M. Dodds W. K. Peterson B. J. (2003). Factors affecting ammonium uptake in streams - an inter-biome perspective. *Freshw. Biol.* 48 1329–1352. 10.1046/j.1365-2427.2003.01094.x

[B135] Welter J. R. Benstead J. P. Cross W. F. Hood J. M. Huryn A. D. Johnson P. W. (2015). Does N2 fixation amplify the temperature dependence of ecosystem metabolism? *Ecology* 96 603–610. 26236857 10.1890/14-1667.1

[B136] West G. B. Brown J. H. Enquist B. J. (1999). The fourth dimension of life: fractal geometry and allometric scaling of organisms. *Science* 284 1677–1679. 10.1126/science.284.5420.1677 10356399

[B137] Williamson T. J. Cross W. F. Benstead J. P. Gíslason G. M. Hood J. M. Huryn A. D. (2016). Warming alters coupled carbon and nutrient cycles in experimental streams. *Glob. Change Biol.* 22 2152–2164. 10.1111/gcb.13205 26719040

[B138] Yvon-Durocher G. Caffrey J. M. Cescatti A. Dossena M. del Giorgio P Gasol J. M. (2012). Reconciling the temperature dependence of respiration across timescales and ecosystem types. *Nature* 487 472–476. 10.1038/nature11205 22722862

[B139] Zhou Z. Guo L. Minor E. C. (2016). Characterization of bulk and chromophoric dissolved organic matter in the Laurentian Great Lakes during summer 2013. *J. Great Lakes Res.* 42 789–801. 10.1016/j.jglr.2016.04.006

